# Supplementary material for: Active site specificity profiling datasets of matrix metalloproteinases (MMPs) 1, 2, 3, 7, 8, 9, 12, 13 and 14
Source: Data Brief. 2016 Feb 22;7:299–310. doi: 10.1016/j.dib.2016.02.036 (PMC4777984; doi:10.1016/j.dib.2016.02.036)
Supplement: Supplementary file 10 — Supplementary material [file mmc10.zip › WebPICS_hMMP14_T_1%/P1prime.html]

 

PICS results


|  |  |
| --- | --- |
| **P1prime\_M**  17 in 307 sites   5.5 %    effects > 10 perc. pnts.  (vice-versa in brackets)  P3\_V: 16.3 (12.7) |  |
| **P1prime\_Q**  14 in 307 sites   4.6 %    effects > 10 perc. pnts.  (vice-versa in brackets)  P3\_S: 21.8 (14.4)   P2\_C: 17.5 (20.4)   P1\_C: 12.0 (24.0)   P2prime\_Q: 27.6 (15.4) |  |
| **P1prime\_V**  34 in 307 sites   11.1 %    effects > 10 perc. pnts.  (vice-versa in brackets)  P2\_N: 13.8 (22.2) |  |
| **P1prime\_W**  12 in 307 sites   3.9 %    effects > 10 perc. pnts.  (vice-versa in brackets)  P1\_G: 57.1 (12.5)   P2prime\_H: 12.5 (11.5) |  |
